# Development of a Novel Bionanocomposite Adsorbent for Adsorptive Separation of Dyestuff from Water

**DOI:** 10.1021/acsomega.5c03133

**Published:** 2025-06-23

**Authors:** Aynur Manzak, Guler Hasirci, Selin Sezen Kina, Nilufer Durmaz Hilmioglu

**Affiliations:** † Faculty of Science, Department of Chemistry, Sakarya University, Esentepe, Serdivan, Sakarya 54050, Turkey; ‡ Faculty of Engineering, Department of Chemical Engineering, 52980 Kocaeli University, Umuttepe, Izmit, Kocaeli 41001, Turkey; § Institute of Science, Department of Environmental Engineering, Kocaeli University, Umuttepe, Izmit, Kocaeli 41001, Turkey

## Abstract

Since malachite green, a dye commonly used in the textile industry, fish farming (as a disinfectant), and trout facility wastewater, is poisonous to all living things, its removal is crucial for the environment and the health of all living organisms. In this study, an environmentally friendly composite bioadsorbent material was synthesized using seaweed-based biopolymer alginate and nanobioglass synthesized from natural materials. The characterization of the developed adsorbent material was performed. Malachite green, a disinfectant and dye causing serious environmental pollution, was adsorbed with the adsorbent developed. The effects of various parameters, including adsorbent amount, dye concentration, time, temperature, and pH, on the adsorption removal rate were investigated. To understand the adsorption behavior, adsorption isotherms, kinetic models, and thermodynamic parameters were examined. It was found that the data obtained through experiments were convenient with the Langmuir isotherm model, the pseudo-first-order kinetic model. As a consequence of the thermodynamic studies conducted with the help of the Van’t Hoff equation, a negative enthalpy difference value showed that the adsorption is exothermic. Negative entropy difference values indicated that physical adsorption occurs through electrostatic interactions. Additionally, the increase in the free energy difference with temperature showed that the probability of adsorption occurring at high temperatures is low. The statistical model analysis showed that the most effective variable on the removal was the time, with the highest *F*-value.

## Graphical abstract

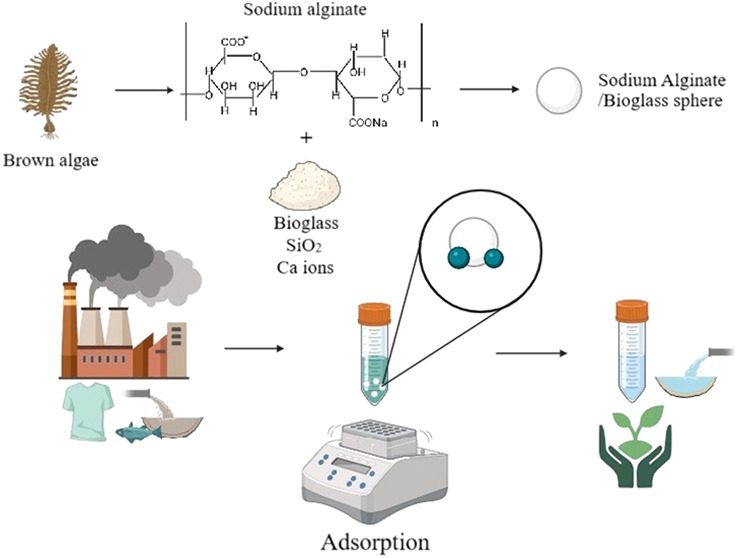

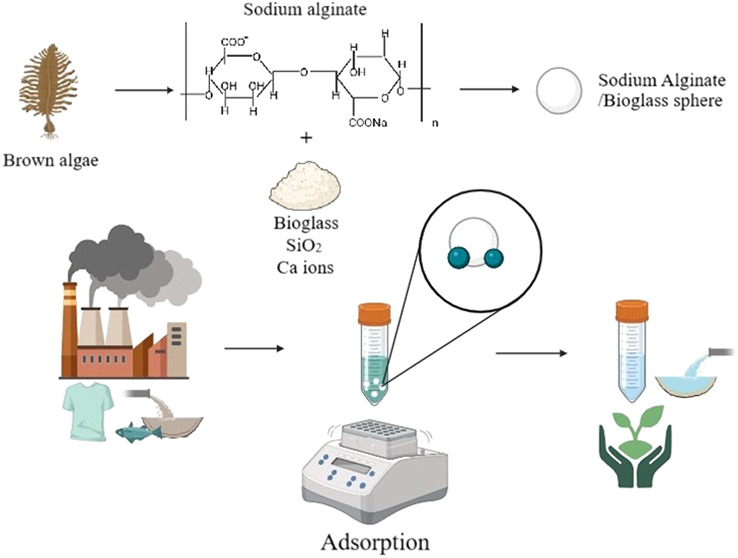

## Introduction

Malachite green, which is used in the textile industry as a dye, is also a disinfectant. It is used for ectoparasitic, fungicidal, and antiseptic purposes in trout farming. Although it is used against bacterial infections in fish, it is extremely toxic to them. Abnormalities were observed in the offspring of fish hatched from eggs treated with malachite green. Furthermore, carcinogenic and mutagenic effects may occur in humans who consume these fish.^1^ Industrial wastewater containing dyes causes serious environmental problems by increasing both the toxicity and the chemical oxygen demand of the wastewater. Most of these dyes are synthetic and have a complex aromatic structure. Dye materials can be carcinogenic and mutagenic. These inert substances are not biodegradable.^2^ Therefore, the separation of dyestuff from industrial processes or wastewater is becoming increasingly important.^3^ Synthetic dyes are utilized in many branches of the industry due to simplicity of use, low cost, stability, and a wide range of colors compared to natural ones. Dyes are generally not biodegradable. The widespread use often causes environmental pollution, particularly through the discharge of colored wastewater into water bodies. This reduces sunlight penetration in streams, thereby limiting photosynthesis. When the color is removed by any method in the wastewater treatment plant, it can pass through the receiving water and cause pollution to the water. New techniques must be developed and implemented to meet the increasing demand for clean water due to the depletion of safe drinking water resources. Various technologies are used to remove dying wastes such as adsorption, membrane technology, extraction, oxidation, ozonation, flotation, and flocculation. However, the removal of such compounds remains a significant problem.

Various industries produce hazardous organic and inorganic wastes. These pollutants, which are extremely harmful to the environment, cannot be treated with classical biological techniques. The primary methods used in the treatment of these pollutants are physical methods, such as flocculation, flotation, filtration, and sedimentation. Secondary methods are aerobic and anaerobic treatment. Tertiary methods are advanced techniques such as adsorption, ion exchange, membrane separation, and oxidation.^4^ Physical, chemical, and biological methods are not effective enough to remove dyes from wastewater. Therefore, advanced oxidation processes are being developed.^5^ Advanced oxidation processes have very high operating costs because they use a lot of energy and chemicals. Therefore, their widespread use is limited.^6^ The advantages of the adsorption process compared to other processes can be listed as follows: it is a low-cost process, easy to install, simple in design, and highly efficient.^7^

Adsorption processes provide an attractive solution, especially when the adsorbent is cheap and accessible. The use of nanoadsorbents may be a solution.^8^ The decrease in water resources and demand for water have further increased the importance of wastewater treatment. Among the methods developed for treating dyes, one of the pollutants in wastewater, adsorption is the most preferred method. The effectiveness and reusability of the adsorbent with a reasonable cost determine the performance of the method.^9^ In the last few decades, adsorption has been carried out using various natural and synthetic adsorbents. However, most of the adsorbents have not been widely applied due to technological or economic limitations.^10^ Therefore, developing effective, low-cost, and natural material adsorbent materials is essential.

In this study, a previously unmade bioadsorbent was synthesized using natural raw materials, and dye removal from model wastewater was investigated by adsorption. Renewable, environmentally friendly biosorbents are attracting attention.^11^ Researchers focus on abundant, renewable, and environmentally friendly marine resources, such as biosorbents. Natural biopolymeric adsorbents have recently attracted great attention due to their low cost, biocompatibility, and environmental friendliness. Among them, polysaccharide-based sodium alginate has taken part in the removal of water pollutants.^10^ Alginate obtained from brown seaweed is a biopolymer with the advantages of lower cost and higher quality through gelation. It is also defined as a green adsorbent.^11^ Alginates extracted from brown algae in seaweed have been defined as superabsorbents. They are polysaccharides consisting of β-(1–4)-d-mannuronic (M) and α-l-gluronic (G) acids.^12^ Biomaterials, such as ceramics, glass, metals, and polymers, are materials with unique properties. Natural polymers are combined with fillers such as glass to improve mechanical performance.^13^ There are reports in the literature on dye removal using adsorbents made by adding inorganic additives to the biopolymer alginate.^14,15^ A new miraculous material has been developed for implant production in biomedical applications using various materials, such as calcium and phosphorus, based on silica (glass). Due to its osteogenic structure, this material has been named bioglass or bioactive glass. Its excellent biocompatibility has positioned it as an innovative material.^16^ Tetraethyl orthosilicate (TEOS), also known commercially as ethyl silica, has the commercial formula Si­(OC_2_H_5_)_4_. Much more material is needed for the synthesis of bioactive glass used in biomedical applications in medicine. Bioglass has extreme biocompatibility, thermostability, a wide surface area, and a nanoscale uniform structure. Because it is degradable in the environment, it can be used without secondary pollution, making it suitable.^3^ Bioglass can be obtained with a few raw materials using the Stöber method.^17^ The purpose of the study is to synthesize bioglass particles, incorporate them into the biopolymer alginate to form composite adsorbents, and investigate their efficiency in dye removal. There are studies in the literature on the preparation of bioactive glass/alginate composites.^18–20^ When these studies are examined, it is understood that bioactive glass synthesis for biomedical applications such as bone prostheses and dental implants varies according to the need, and the synthesis conditions are multistep, long, and require a lot of material. There is no need to use bioactive glass in nonmedical applications, and the use of bioglass in the adsorption process seems more appropriate. In bioglass synthesis, it is more economical to choose simple synthesis conditions, such as the Stober method. Bioglass is used in powder form as an adsorbent. However, in this form, the separation of the adsorbent from the system after adsorption requires additional processes and results in material loss. Using bioglass as a composite with alginate, a biocompatible biopolymer-like itself, in the production of adsorbents allows for reuse. Bioglass is used as a catalytic support, adsorbent, and catalyst and has superior surface properties compared to zeolites and clays. Due to the negative charges on their surfaces, bioglass can be considered a suitable adsorbent for cationic dyes. However, the use of bioglass in powder form limits its recovery and reusability.^10^ Designing composite adsorbents and incorporating bioglass into a biopolymer solve this problem. Adsorption with bioglass alone is also available in the literature.^2^ In addition, adsorption studies with membranes using alginate and bioglass materials have been seen in the literature.^10^ In these studies, methylene blue removal by adsorption was made. The difference in this study is that the adsorbent is in the form of a spherical bead. Furthermore, the adsorption of the cationic dye malachite green, which has not been studied before in the literature, was studied in this study.

There are adsorption studies in the literature on bioactive glass powder. Bioactive glass is applied in the medical field. The substances (B_2_O_3_, Na_2_O, CaO, and P_2_O_5_) and the preparation conditions used in the synthesis of bioactive glass used in copper removal from water by adsorption are different.^21^ In the removal of methylene blue dye with La- and Ti-doped bioactive glass, the bioactive glass synthesis conditions and raw materials (SiO_2_, CaO, P_2_O_5_, La (NO_3_)_3_ 6H_2_O, C_16_H_36_O_4_Ti) are again different.^22^ Dyestuff removal with pure alginate is also available in the literature.^23^ Activated carbon/clay/sodium alginate composite hydrogel membranes have been used in dye adsorption.^24^ Dyestuff removal was studied with an alginate/titania nanoparticle composite.^25^ As seen, dye removal was studied with alginate composites. Dye removal was also studied with bioactive glass in powder form. Dye removal was not studied with bioglass prepared with the recipe used in this study. While more material is used in bioactive glass synthesis, much less material (TEOS, calcium nitrate tetrahydrate) is used in bioglass synthesis, which shows the originality of the study. It was seen in the literature that methylene blue removal with the film form (membrane) prepared with bioglass-added alginate.^10^ The difference and innovation of this study is that bioglass-added alginate adsorbent was prepared in a sphere form to remove malachite green from water for the first time.

## Materials and Methods

### Materials

Sodium alginate, TEOS (>97,5%), calcium nitrate tetrahydrate (>99.0%), and CaCl_2_ (>97.0%) were purchased from ISOLAB. Ammonia solution (30%) was purchased from CARLO ERBA. Ethanol (absolute), HCl (37%), NaOH, and NaCl were purchased from MERCK. Malachite green was purchased from Kimyalab.

### Preparation of Biocomposite Adsorbent Beads

Bioglass is prepared according to the Stober Method in the literature. Ethanol and ammonium hydroxide aqueous solutions and TEOS ethanol solutions are mixed. Calcium nitrate tetrahydrate is added to the mixture. The resulting suspension is centrifuged. The resulting slurry is rinsed with water and an ethanol solution. The remainder is then dried.^10,17,26^ To prepare a biopolymer alginate solution at a certain concentration, sodium alginate is dissolved in hot water. A suspension consisting of bioglass with a small amount of water is sonicated in an ultrasonic bath. The resulting suspension is added to the biopolymer solution using the priming technique. The viscous solution that contains a certain amount of bioglass is dropped into the coagulation bath consisting of CaCl_2_, and biocomposite adsorbent spheres are formed by mixing them for 24 h in the bath. The resulting spheres are washed with water until they become neutral and then dried at room temperature.^27^ Bioglass was added to alginate at rates of 5, 10, and 15%. Adsorption efficiency was low for the adsorbent containing 5% bioglass, while agglomeration occurred in the 15%. Therefore, the process was continued using the adsorbent with 10% bioglass.

### Batch Adsorption Studies

A model dye solution was prepared for the adsorption process. A malachite green stock solution was prepared by dilution. The adsorption process reached equilibrium in 180 min. Adsorption experiments were conducted over 180 min at different concentrations of 10, 15, and 20 mg/L at the pH of the dyestuff solution for adsorbent amounts of 30, 40, and 50 mg, at shaking speed 300 rpm. The maximum wavelength of the dye solution is 617 nm in the UV–vis spectrophotometer. The adsorption efficiency for removal is determined by eq 1 ^28^

1 $$\mathrm{removal efficiency}\;\left(\%\right)=\left(C_{0}-C\right)\times 100/C_{0}$$

*C*_0_ and *C* are the initial concentrations measured before adsorption and the equilibrium concentrations measured after adsorption.

The adsorption capacity, representing the amount of malachite green adsorbed (mg/g), was calculated using eq 2 ^27^

2 $$q_{e}=\left(\left(C_{0}-C_{e}\right)/m\right)\times V$$

*C*_0_ and *C*_e_ are the concentrations measured before and after the experiment (mg L^–1^), *V* is the volume of the dyestuff solution (L), and *m* is the amount of the adsorbent (g).

### Isotherm, Kinetic, and Thermodynamic Studies

Adsorption isotherm studies determine the maximum capacity of adsorption by examining the relationship between the adsorbate and the bioadsorbent.^29^ The isotherm models examine the equilibrium state between the bioadsorbent and the adsorbate. It determines the relation between *C*_e_ and *q*_e_ at the equilibrium. The experimental data were analyzed using several isotherm models, including Langmuir, Freundlich, Temkin, and Dubinin–Radushkevich. Adsorption kinetics models examine the rate and time of adsorption and the adsorbate.^29^ The kinetic models examine the mass transfer mechanism in the adsorption process. The kinetic parameters of dye on the surface of the adsorbent are determined. The experimental data were analyzed using kinetic model equations such as pseudo-first order, pseudo-second order, Elovich, and Weber–Morris intraparticle diffusion. The temperature effect for the adsorption effectiveness was investigated with the help of the Van’t Hoff equation.^30^ The adsorption process is examined thermodynamically by calculating the enthalpy change, entropy change, and distribution coefficient.

### Statistical Analysis

The response surface method (RSM) examines the effects of independent variables, called factors, on a response. Optimization by minimizing cost and time was performed using either the Box–Behnken method or the central composite design method. The main stages of RSM include reducing the number of experiments, determining independent variables that have effects on the response, and investigating the optimum point.^31^

## Results and Discussions

### Characterization of the Adsorbents

FTIR analysis is given in Figure 1.

**Figure 1:**
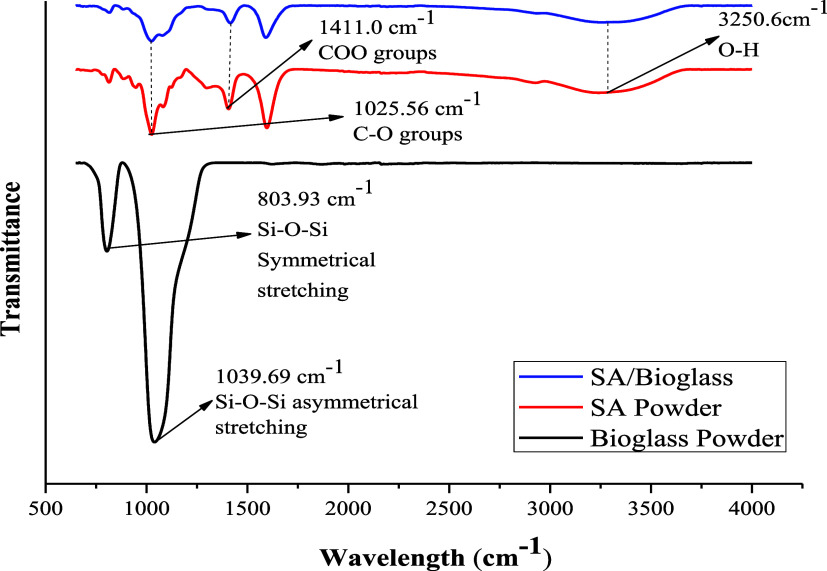
FTIR spectra of bioglass, sodium alginate (SA), and SA/bioglass.

FTIR characterization of the produced bioglass and SA and SA/bioglass is shown in Figure 1. Peaks at 3250.6, 1411.0, and 1025.56 cm^–1^ wavelengths are associated with OH, COO, and CO groups in SA and SA/bioglass structures, respectively.^32^ The peaks at 1039.69 and 803.93 cm^–1^ in bioglass are Si–O–Si asymmetric and symmetric modes that are stretching.^3,33^ TGA analysis is listed in Figure 2.

**Figure 2:**
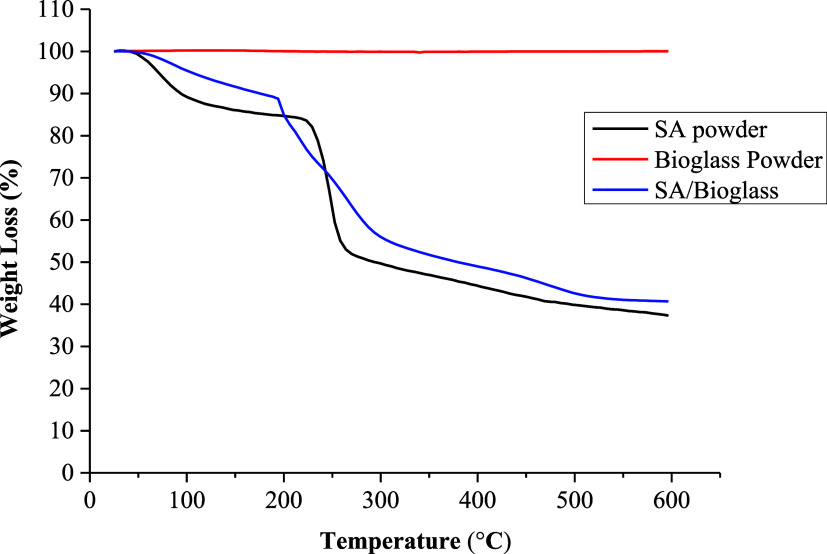
TGA curves of bioglass, sodium alginate (SA), and SA/bioglass.

TGA curves of SA, bioglass powder, and SA/bioglass samples between 25 and 600 °C are shown in Figure 2. For SA, two thermal degradation regions were observed. The initial thermal degradation between 50 and 230 °C is due to the removal of moisture from the alginate structure, while above 230 °C, where large mass loss occurs, the alginate components are decomposed.^34,35^ In the TGA curve of bioglass powder, the thermal stability of the bioglass structure reaches its maximum due to the calcination process, with no mass loss observed between 25 and 600 °C.^36^ At the end of the analysis, since the residual amount of the SA/Bioglass sample was higher than SA, the participation of bioglass in the sodium alginate structure increased the thermal stability of the structure.^35^ XRD analysis is given in Figure 3.

**Figure 3:**
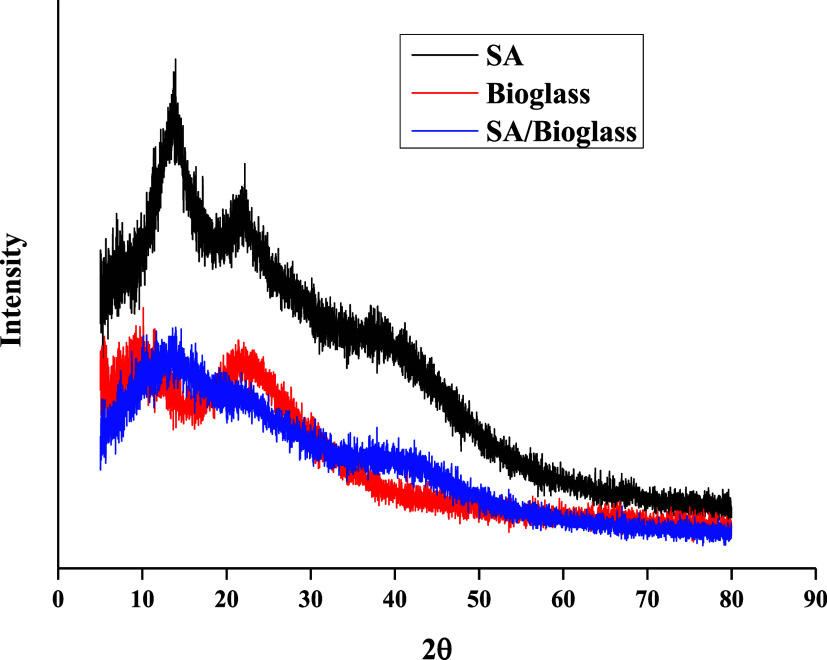
XRD analysis of bioglass, sodium alginate (SA), and SA/bioglass.

XRD analysis of the produced bioglass, SA, and SA/bioglass is shown in Figure 3. The peaks of diffraction observed at 2θ = 13 and 23° for pure sodium alginate originate from polyguluronate and polymannuronate units, indicating the semicrystalline structure of sodium alginate. The 2θ = 39° diffraction peak represents the amorphous halo of sodium alginate.^37,38^ The broad diffraction peak observed at 2θ = 23° in the bioglass sample is not sharp, indicating that the bioglass is amorphous.^39,40^ The SA/bioglass sample showed a reduction in the intensity of the diffraction peak that is 2θ = 13°. Bioglass added to the SA structure shifted the semicrystalline structure to an amorphous side.^41^ SEM analysis is given in Figure 4, and EDX analysis is presented in Figure 5.

**Figure 4:**
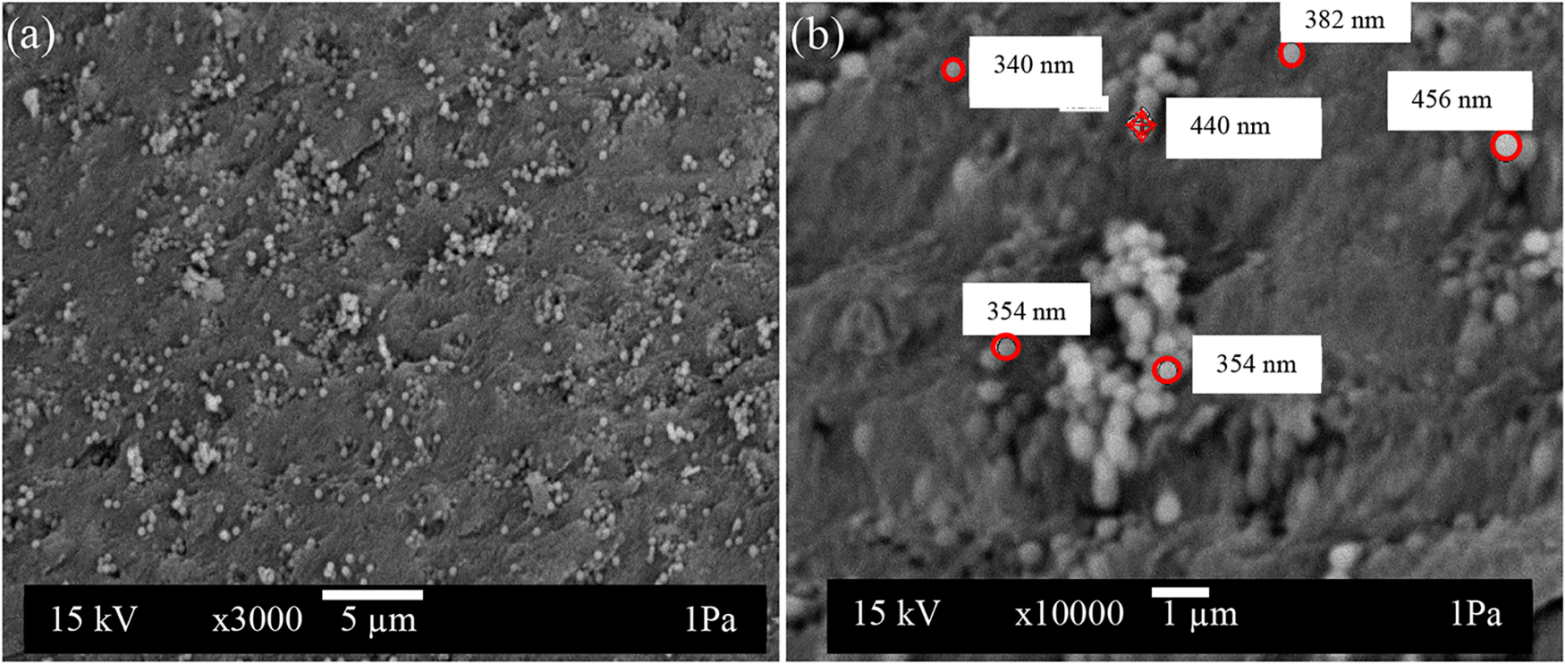
SEM analysis of bioglass in the SA structure: (a) bioglass (×3000) and (b) bioglass (×10,000).

**Figure 5:**
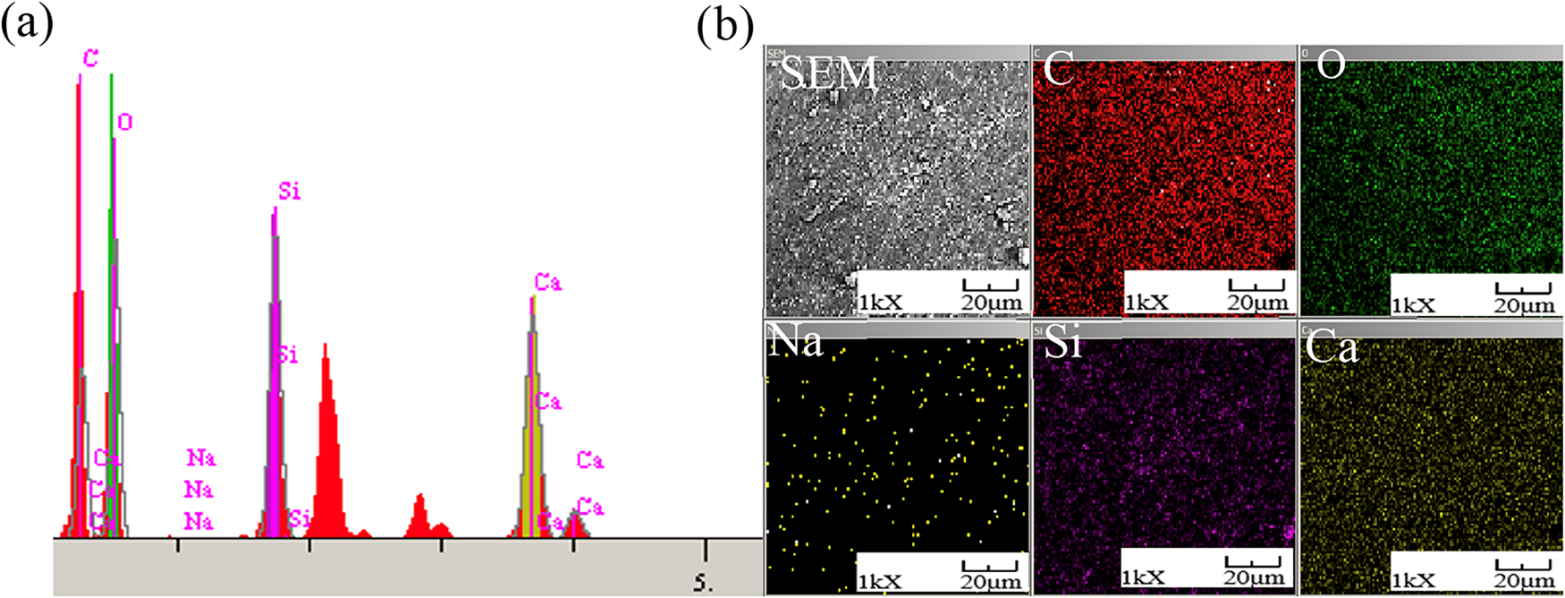
EDX analysis of bionanocomposite spheres, (a) elemental analysis, and (b) elemental mapping.

The SEM image revealed that the bioglass grains were homogeneously distributed in the alginate biopolymer, and bioglass spheres ranged in size from 340 to 462 nm.

According to the calculation by EDX analysis, the bioglass-loaded sodium alginate adsorbent spheres contain 16.837% Ca, 12.692% Si, 0.109% Na, 47.480% O, and 22.883% C.

### Effects of Process Variables

The concentrations of Malachite green dye solution used in adsorption tests were 10, 15, and 20 mg/L. Adsorbent amounts of 30, 40, and 50 mg were used. Tests were made at 25 °C and the dye’s own pH value of the dye solution for 20 mL of dye solution. Since the efficiency decreased at the end of the 180 min adsorption period, adsorption tests were conducted for 180 min.

As seen in Figure 6, because of the increase in the adsorption centers in the adsorbent, adsorption removal rates increased with increasing adsorbent amount in all dye concentrations.

**Figure 6:**
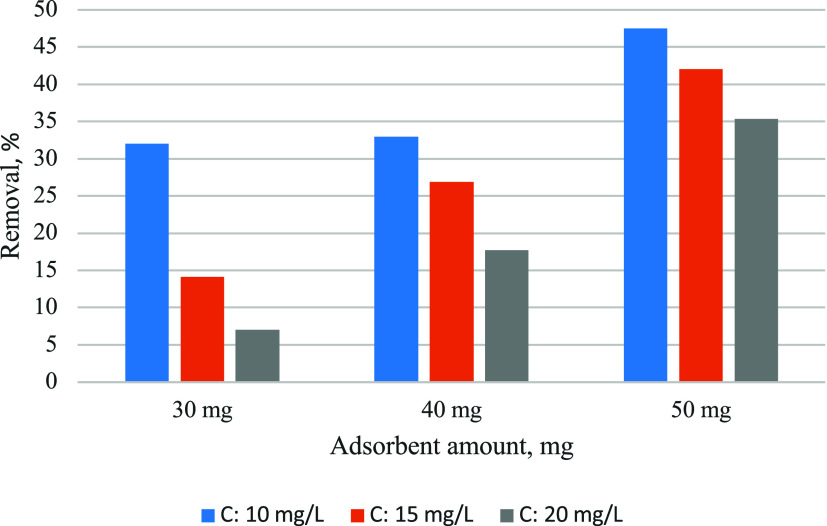
Effect of adsorbent amount on adsorption efficiency (25 °C).

The effect of the dye concentration is seen in Figure 7. Adsorption removal rates decreased with increasing dye concentration in all adsorbent amounts as a result of the decrease of the adsorption centers in the adsorbent by filling with dye.

**Figure 7:**
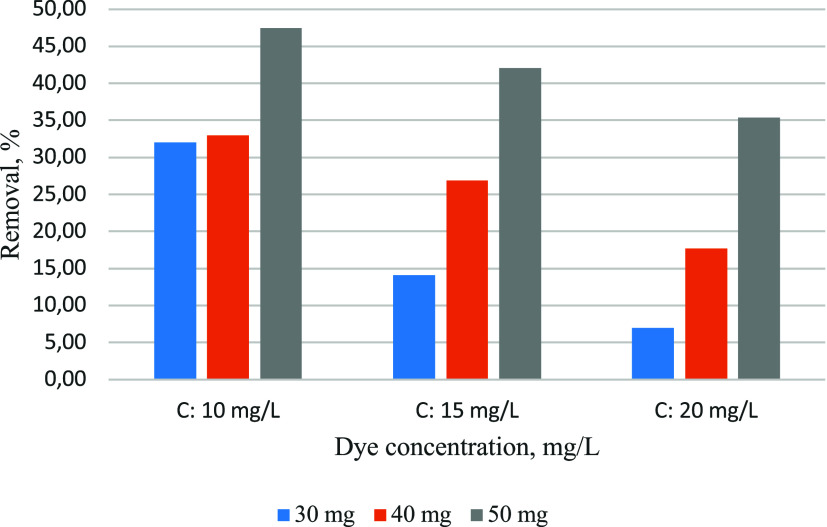
Effect of the dye solution concentration on the adsorption efficiency (25 °C).

The pH value of the zero charge point (pH_zpc_) was found experimentally. The pH values tested were 1, 3, 6, and 12. The pH was set with dilute 0.1 N HCl and 0.1 N NaOH. 20 mL of 0.01 M NaCl solution was placed into 50 mL containers, and the pH was adjusted to target values (1, 3, 6, 12). The first measured pH was called pH_1_. Then, 50 mg of adsorbent was added to each container. The obtained solutions were mixed at room temperature for 24 h, after which the final pH was measured and recorded as pH_2_.

pH_1_ – pH_2_ were calculated, and the pH value at the zero charge point was found from the graph drawn against pH_1_ for the pH difference that is pH_1_ – pH_2_.^42^

In Figure 8, the value of pH_zpc_ is found from the point where the curve intersects the horizontal axis. The pH of the paint was 6.46, while the zero point of charge was found to be 6.15. Therefore, adsorption was performed for the original pH value of the dye, which is also economically favorable. It is economical to work at this pH value. Since the pH value of the dye is higher than the pH value of the zero charge point, it was studied in the negative region. The dye has a positive property. Due to electrostatic attraction, OH^–^ ions attracted dye^+^ ions. In the removal of malachite green, a cationic dye, with the developed anionic adsorbent, adsorption is expected to occur at basic pH values greater than the pH value at the zero charge point. At these values, negatively charged OH^–^ ions also attract positively charged dye molecules. Adsorption is not expected to occur at acidic pH values smaller than the pH value at the zero charge point. This is because positively charged H^+^ ions repel positively charged dye molecules.^43^

**Figure 8:**
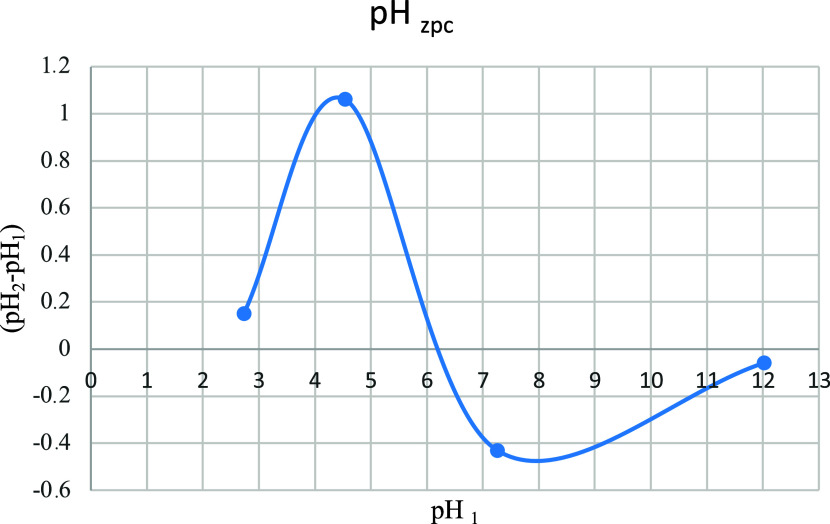
Zero point charge pH value.

The effect of pH on adsorption efficiency was given as removal in Figure 9a for a dye concentration of 10 mg/L, an adsorbent amount of 50 mg, and a temperature of 25 °C for 180 min.

**Figure 9:**
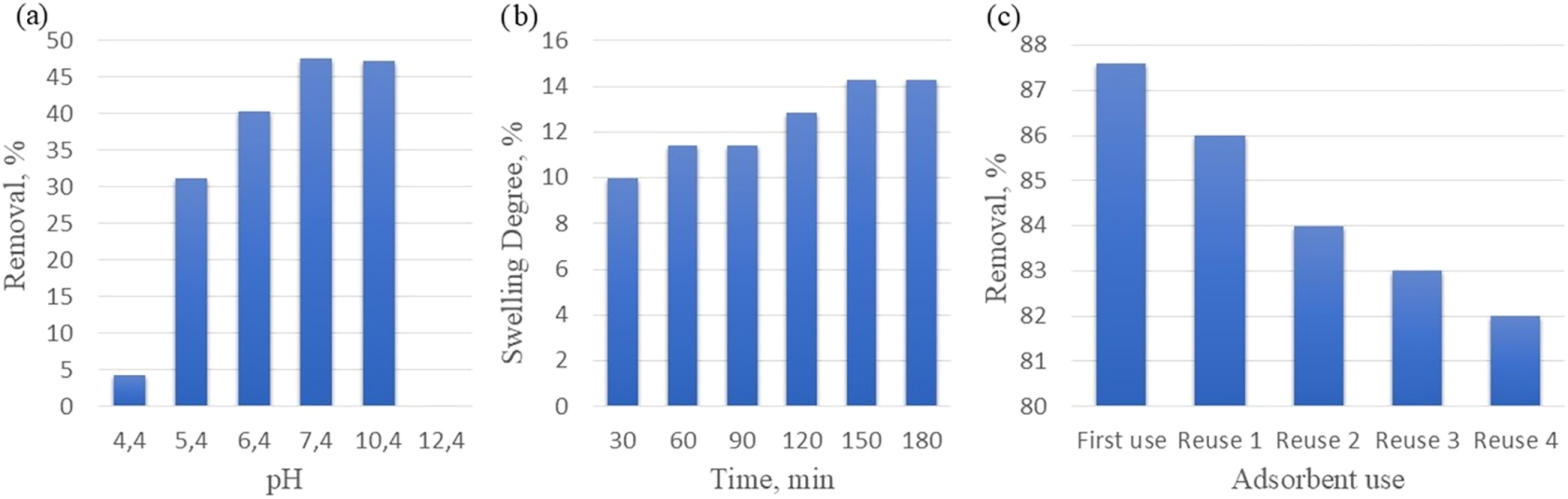
(a) Effect of pH, (b) Swelling degrees, and (c) Reuse potential.

The dye solution is resistant to almost all pH values and dissolves under only highly basic conditions. The adsorbent dissolved at pH 12. The removal is low at acidic pH values and high at basic pH values. Because of cost, the removal obtained at the pH value of the dye and the removal obtained under basic conditions are close to each other, so the dye’s own pH value was studied.

Swelling tests were performed with pure water at 25 °C. The degree of swelling was calculated as seen in eq 3. Sorption results of the adsorbent as swelling degrees are given in Figure 9b.

3 $$s\mathrm{welling degree}\;\left(\%\right)=\left[\left(m_{\mathrm{wet}}-m_{\mathrm{dry}}\right)/m_{\mathrm{dry}}\right]\times 100$$

The degree of swelling reached a constant value after 180 min, which was also the adsorption time.

The reuse potential of the adsorbent has been evaluated. The reuse of the adsorbent was determined by adsorption–desorption cycle tests and is given in Figure 9c. Regeneration was done by using an ethanol solution with NaOH.^2^ Reuse tests were done for a dye concentration of 10 mg/L, an adsorbent amount of 50 mg, an adsorption temperature of 25 °C, a desorption temperature of 45 °C, and a time of 120 min, shaking speed 600 rpm as shown in Figure 9c. It has been observed that the developed adsorbents can be reused. The fact that the removal rates are at the same levels in reuse points to the importance of using nanomaterials.

### Adsorption Mechanism

Alginate is negatively charged,^44^ and the bioglass surface is also negatively charged.^2,26^ There are electrostatic interactions between the negative charges of the bioadsorbent and the positive charges of the dyestuff. In addition, hydrogen bonds are formed with the uncross-linked OH^–^ groups of the alginate and the free Si–O– group in the bioglass. Adsorption occurs with the interaction of electrostatic interaction forces and hydrogen bonds.^10,45^

The adsorption mechanism was also explained by measuring the ζ-potential value and finding the zero charge point with pH experiments.

The ζ-potential of the SA/bioglass was measured at −23.77 mV, which shows that the particles were unstable in water and can act as an adsorbent, especially for cationic molecules via electrostatic interactions. Malachite green is a cationic dye; therefore, the ζ-potential was expected to increase after adsorption. As expected, after keeping for 1 h in malachite green solution, the sample ζ-potential was increased to −17.72 mV due to the adsorbed malachite green on the sample. For adsorption for 2 h, even ζ-potential continued its increasing trend, which shows that adsorption is ongoing.

The adsorption mechanism is also explained in detail as follows:

Adsorption occurs through an electrostatic interaction between the adsorbent and the dye. ζ-potential is a property of the adsorbent that shows this interaction. The isoelectric point is determined by ζ-potential measurements at different pH values. The isoelectric point is defined as the point of zero charge.^46^

In this study, the point of zero charge (pH_zpc_) was found with the pH values measured before and after the adsorption experiments with salt solutions at different pHs and is shown in Figure 8. The original pH value of the dye used in the study is greater than the pH value at the point of zero charge and corresponds to the negative region in the graph. This situation allows electrostatic interaction with the positive dye.

In addition, the measured ζ-potential value was −23.77 (mV). Thus, it was proven that the adsorbent was negative. Adsorption occurred with the electrostatic interaction of the negative bioglass-added alginate adsorbent with the positive dye malachite green. The negative ζ-potential of the adsorbent at the pH_zpc_ value allows it to attract the oppositely charged dye.

### Isotherm Studies

The dyestuff concentration at equilibrium and the adsorptive capacity of the bioadsorbent were examined with isotherm models. The mathematical equations of isotherm models^47^ and the related results are given in Table 1.

**Table 1:** Isotherm Equations with Parameters and Regression Coefficients

isotherm	equation	parameters, adsorbent: 50 mg, *C*_0_: 10 mg/L	regression coefficient for different concentrations
Langmuir	1/*q*_e_ = 1/*q*_m_ + 1/(*bq*_m_ *C*_e_)	*K*_L_ = 1/(1 + *bC*_0_)	*R*^2^ = 0.9909 (10 mg/L)
		*K*_L_ = 0.403 (*C*_0_: 10 mg/L)	*R*^2^ = 0.9878 (15 mg/L)
		*q*_m_ = 4.38	*R*^2^ = 0.9817 (20 mg/L)
Freundlich	log *q*_e_ = log *K*_F_ + (1/*n*) log *C*_e_	*K*_F_ = 0.927	*R*^2^ = 0.9696 (10 mg/L)
		*n* = 2.254	*R*^2^ = 0.9615 (15 mg/L)
			*R*^2^ = 0.9545 (20 mg/L)
Temkin	*q*_e_ = *B*_T_ ln *K*_T_ + *B*_T_ ln *C*_e_	*B*_T_ = 1.029	*R*^2^ = 0.9832 (10 mg/L)
	*B*_T_ = *RT*/*b*	*K*_T_ = 1.242	*R*^2^ = 0.9725 (15 mg/L)
			*R*^2^ = 0.9697 (20 mg/L)
Dubinin–Radushkevich	ln *q*_e_ = ln *q*_m_– *K*_DR_ε^2^	*K*_DR_ = 3 × 10^–6^	*R*^2^ = 0.9889 (10 mg/L)
	ε = *RT* ln[1 + 1/*C*_e_]	*q*_m_: 3.060	*R*^2^ = 0.9819 (15 mg/L)
	*E* = (1/√2*K*_DR_)	*E* = 0.408 kJ/mol	*R*^2^ = 0.9776 (20 mg/L)

*q*_e_ is the adsorptive capacity (mg/g), *C*_e_ is the equilibrium concentration at (mg/L), respectively, *K*_L_ is the Langmuir constant, *q*_m_ is the maximum adsorptive capacity of the bioadsorbent (mg/g), *b* is the constant, and *C*_0_ is the initial adsorbate concentration (mg/L). *K*_F_ is the Freundlich constant, and n is the constant. *B*_T_ is the Temkin constant, and *b* is the constant. The numerical value of *R*, that is, the gas constant is 8.314 (J mol/K), *T* is the temperature (K), and *K*_T_ is the binding constant at the equilibrium. *K*_DR_ is the Dubinin–Radushkevich constant; ε defines the Polanyi potential, and *E* is the average of the adsorption energy. Since the regression coefficient was the biggest, it was observed that the studied data were compatible with the Langmuir isotherm that is a monolayer and homogeneous adsorption process occurs.^47^ In the Dubinin–Radushkevich isotherm, when *E* is between 8 and 16 kJ mol^–1^, there is ion exchange during adsorption; when *E* < 8 kJmol^–1^, there is physical adsorption; and when 20 < *E* < 40 kJmol^–1^, there is chemical adsorption.^48^ According to the calculated *E* value, the adsorption process is a physical process.

### Kinetic Studies

The change in the adsorption capacity over time was examined with kinetic models. The mathematical equations of kinetic models^47^ and the related results are given in Table 2.

**Table 2:** Kinetic Equations with Parameters and Regression Coefficients

kinetic	equation	parameters, adsorbent: 50 mg, *C*_0_: 10 mg/L	regression coefficient for different concentrations
pseudo-first order	log(*q*_e_ – *q_t_*) = log *q*_e_ – (*k*_1_/2.303)*t*	*k*_1_ = 0.0218	*R*^2^ = 0.9752 (10 mg/L)
		*q*_e_ theoretical: 3.6567	*R*^2^ = 0.9702 (15 mg/L)
		*q*_e_ experimental: 1.9	*R*^2^ = 0.9613 (20 mg/L)
pseudo-second order	*t*/*q_t_* = 1/(*k*_2_ *q*_e_^2^) + *t*/*q*_e_	*k*_2_ = 1.644 × 10^–3^	*R*^2^ = 0.6508 (10 mg/L)
		*q*_e_ theoretical: 0.0169	*R*^2^ = 0.6413 (15 mg/L)
		*q*_e_ experimental: 1.9	*R*^2^ = 0.6401 (20 mg/L)
Elovich	*q*_t_ = 1/β ln(α·β) + 1/β ln(*_t_*)	α = 0.0394	*R*^2^ = 0.9648 (10 mg/L)
		β = 0.9805	*R*^2^ = 0.9611 (15 mg/L)
			*R*^2^ = 0.9544 (20 mg/L)
Weber–Morris particle intra-diffusion	*q*_t_ = *k*_id_·*t* ^1/2^ + *c*	*k*_id_ = 0.1958	*R*^2^ = 0.9316 (10 mg/L)
		*c* = −0.63	*R*^2^ = 0.9302 (15 mg/L)
			*R*^2^ = 0.9276 (20 mg/L)

*q*_e_ is the adsorption capacity at the equilibrium (mg/g), *q_t_* is the time-dependent adsorptive capacity at any time (mg/g), *k*_1_ (min^–1^) is the pseudo-first-order kinetic model constant, and *k*_2_ (g·mg^–1^·min^–1^) is the pseudo-second-order kinetic model constant. α (mg·g^–1^·min^–1^) is the primary adsorption speed constant of the Elovich kinetic model, and β (mg·g^–1^) is the constant that represents the surface coverage size of the layer on the adsorbent. *k*_id_ (mg·g^–1^·min^–0.5^), Weber–Morris interparticle diffusion model constant, and c represents the surface thickness between the adsorbate and the adsorbent. Since the pseudo-first-order kinetic model has the highest regression coefficient, the studied data were found to be compatible with this model. In this model, experimental and theoretical *q*_e_ values are also close to each other.

### Thermodynamic Studies

The thermodynamic studies investigate the feasibility of adsorption thermodynamically. The thermodynamic equations^47^ and the related results are given in Table 3.

**Table 3:** Thermodynamic Expressions and Estimated Values

			Δ*G*° (kJ/mol)		
thermodynamic equations	Δ*H*° (kJ/mol)	Δ*S*° (kJ/mol K)	298 K	308 K	318 K
Δ*G*° = Δ*H*° – *T*Δ*S*°	–97.523	–0.330	2.876	5.825	9.257
Δ*G*° = −*RT* ln *K*_c_					

The equilibrium constant of adsorption (*K*_c_) was calculated from eq 4 and Van’t Hoff equation in eq 5

4 $$K_{c}=q_{e}/C_{e}$$

5 $$\ln K_{c}=\left(\Delta S^{0}-\Delta H^{0}/T\right)\times 1/R$$

*K*_c_ is the adsorption equilibrium constant (g/L), *q*_e_ is the adsorption capacity at equilibrium (mg/g), and *C*_e_ is the concentration at equilibrium (mg/L). Δ*G* ^0^ is the difference of free energy (kJ/mol), Δ*H* ^0^ is the difference of enthalpy (kJ/mol), Δ*S* ^0^ is the difference of entropy (kJ/mol K), *T* is the absolute temperature (K), and *R* is the ideal gas constant (*R*: 8.314 J/(mol K)).

Δ*H* ^0^ is found from the slope obtained by drawing the ln *K*_c_ versus 1/*T*, and Δ*S* ^0^ is determined from the intercept as seen in Figure 10 of Van’t Hoff graph.

**Figure 10:**
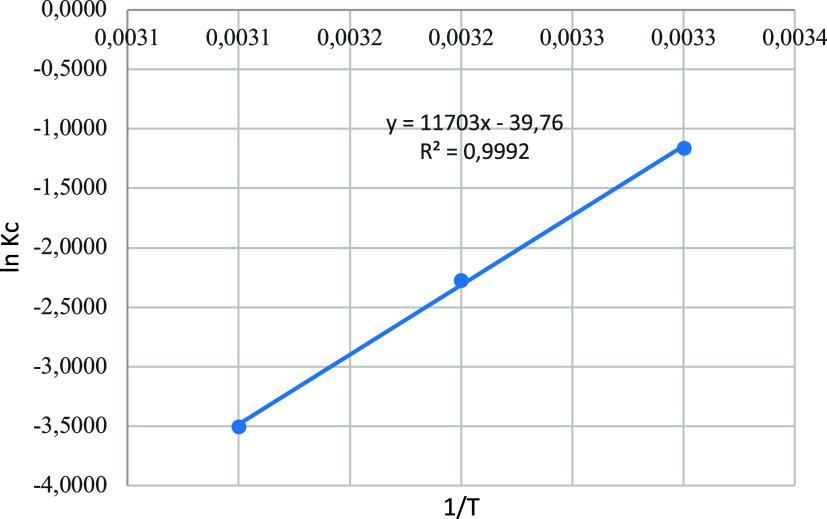
Van’t Hoff equation graph.

The negative Δ*H* ^0^ value points to the adsorption being exothermic and can be applied spontaneously. The negative Δ*S* ^0^ value points to perturbing at the solid/liquid interface decreases while adsorption continues.^49^ Negative Δ*S* ^0^ values indicate a decrease in the adsorbate concentration at the solid–solution interface. In other words, the adsorbate concentration on the adsorbent increases. This indicates that physical adsorption occurs via electrostatic interactions.^50^ The increase in Δ*G* ^0^ by temperature shows that the feasibility of adsorption is lower at higher temperatures.^51^

### Fitting Model and Optimization by RSM

Statistical analysis of the study and the effect of the studied parameters (adsorbent amount, dye concentration, and time) on the removal were evaluated by the RSM study. The actual and coded values of the adsorption variables in Box–Behnken are given in Table 4.

**Table 4:** Actual and Coded Values of Adsorption Variables

			levels		
variables	symbols	units	–1	0	1
adsorbent amount	A	mg	30	40	50
concentration	B	mg/L	10	15	20
time	C	min	60	120	180

When the specified variable values were entered into the program, 17 experiments were proposed by the Box–Behnken design. According to the removal rates in these experiments, the most appropriate model for the data, as specified by the program, is the linear model shown in Table 5. A *p*-value of <0.0001 for the linear model indicates that the model is appropriate for the data.

To verify the adequacy of the model fit, model significance tests between variables are given in Tables 5–7. In Tables 5 and 6, where the linear model is compared with other models, the linear model is recommended by the program with the smallest *p* value (<0.0001), the highest predicted *R* ^2^, and the lowest press value (787.58). Thus, this was the most significant model for the data.

**Table 5:** Fit Summary

source	sequential *p*-value	lack of fit value	adjusted *R* ^2^	predicted *R* ^2^	
linear	<0.0001		0.7519	0.5883	suggested
2FI	0.1113		0.8184	0.4596	
quadratic	0.3746		0.8291	–0.1960	
cubic			1.0000		aliased

**Table 6:** Model Summary Statistics

source	std. dev.	*R* ^2^	adjusted *R* ^2^	predicted *R* ^2^	press	
linear	5.45	0.7984	0.7519	0.5883	787.58	suggested
2FI	4.66	0.8865	0.8184	0.4596	1033.83	
quadratic	4.52	0.9253	0.8291	–0.1960	2288.10	
cubic	0.0000	1.0000	1.0000			aliased

In addition, a *p*-value of <0.0001 in Table 7 indicates that the linear model is more appropriate for the data.

**Table 7:** Sequential Model Sum of Squares

source	sum of squares	df	mean square	*F*-value	*p*-value	
mean vs total	5955.96	1	5955.96			
linear vs mean	1527.54	3	509.18	17.17	< 0.0001	suggested
2FI vs linear	168.52	3	56.17	2.59	0.1113	
quadratic vs 2FI	74.10	3	24.70	1.21	0.3746	
cubic vs quadratic	143.01	3	47.67			aliased
residual	0.0000	4	0.0000			
total	7869.12	17	462.89			

In the ANOVA analysis presented in Table 8, the effect sizes of the adsorption variables on the removal rate can be analyzed. The *p*-values for the three variables are less than 0.05, indicating that the studied parameters have a significant effect on the removal rate. The most effective variable for removal is the time variable with the highest *F*-value (19.60). The fact that all variables are statistically significant with *p* values less than 0.05 also means that the data are within the 95% confidence interval. This shows that the model is appropriate for the experimental data.^27^

**Table 8:** ANOVA for Linear Model

source	sum of squares	df	mean square	*F*-value	*p*-value
model	1527.54	3	509.18	17.17	<0.0001
*A*	403.28	1	403.28	13.60	0.0027
*B*	542.85	1	542.85	18.30	0.0009
*C*	581.40	1	581.40	19.60	0.0007
residual	385.62	13	29.66		
lack of fit	385.62	9	42.85		
pure error	0.0000	4	0.0000		
cor total	1913.16	16			

The fit statistics of the data entered into the program are given in Table 9. Since the difference between the estimated *R* ^2^ value (0.5883) and the adjusted *R* ^2^ value (0.75191) is less than 0.2, the model is significant. Adequate precision must be greater than 4. The adequate precision of 12.6898 indicates that the model can be used for the design.

**Table 9:** Fit Statistics

std. dev.	5.45	*R* ^2^	0.7984
mean	18.72	adjusted *R* ^2^	0.7519
C.V., %	29.10	predicted *R* ^2^	0.5883
		adeq precision	12.6898

The final equation used factors is indicated in eq 6.

6 removal(%)=18.72+7.10A−8.24B+8.53C

Figure 11 shows the residual plots of the model. Residuals represent the difference between experimental and predicted values.^52^ Normally, this difference should be small for the model to be appropriate. The normal probability plot in Figure 11a shows that the data are normally distributed with a reasonable clustering of the data around the red line.^53^ The random distribution of the data in the residuals versus the predicted values given in Figure 11b, without a specific pattern around the line passing through zero, indicates that the data are suitable for the model. In addition, the model has a constant variance of the original observation.^54^ In Figure 11c, the random distribution of the data in the number of runs versus residual values indicates the adequacy of the model.^55^

**Figure 11:**
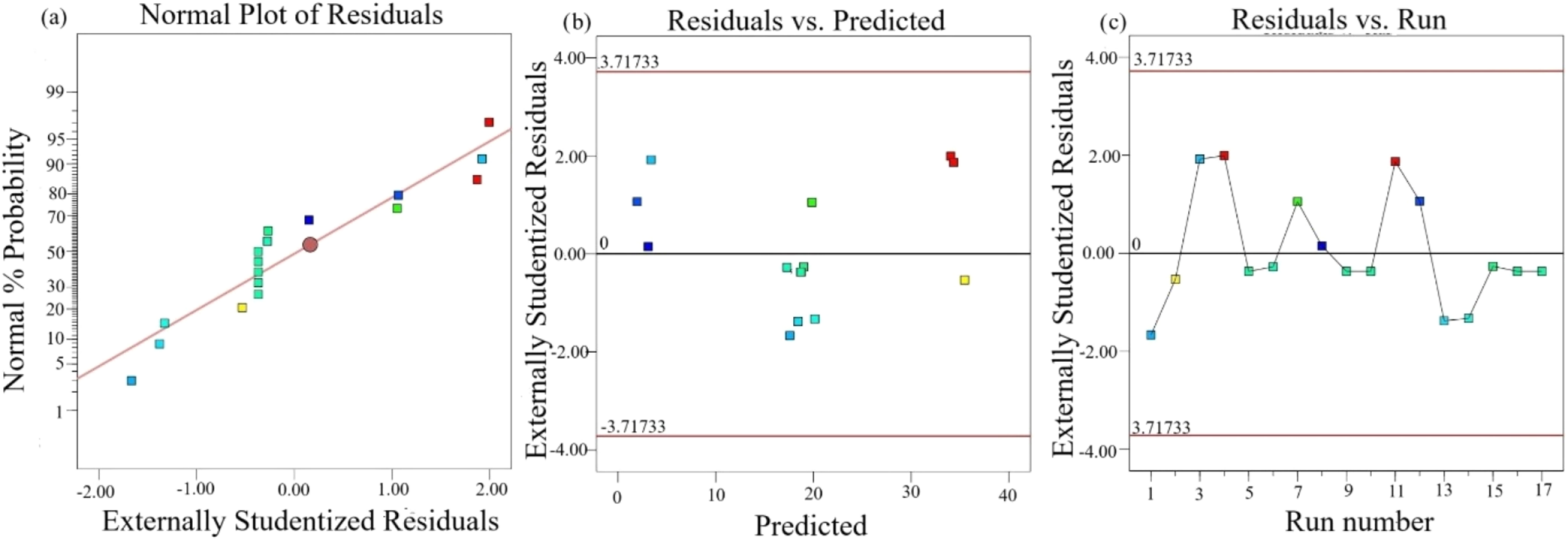
Residual plots: (a) Normal plots of residuals, (b) residual vs predicted, and (c) residuals vs run.

The closeness of the predicted and actual removal data is shown in Figure 12. The actual and estimated values of the removal rates approaching the *y* = *x* line are close. Thus, this indicates that the usability of the experimental design of the model for optimization has improved.

**Figure 12:**
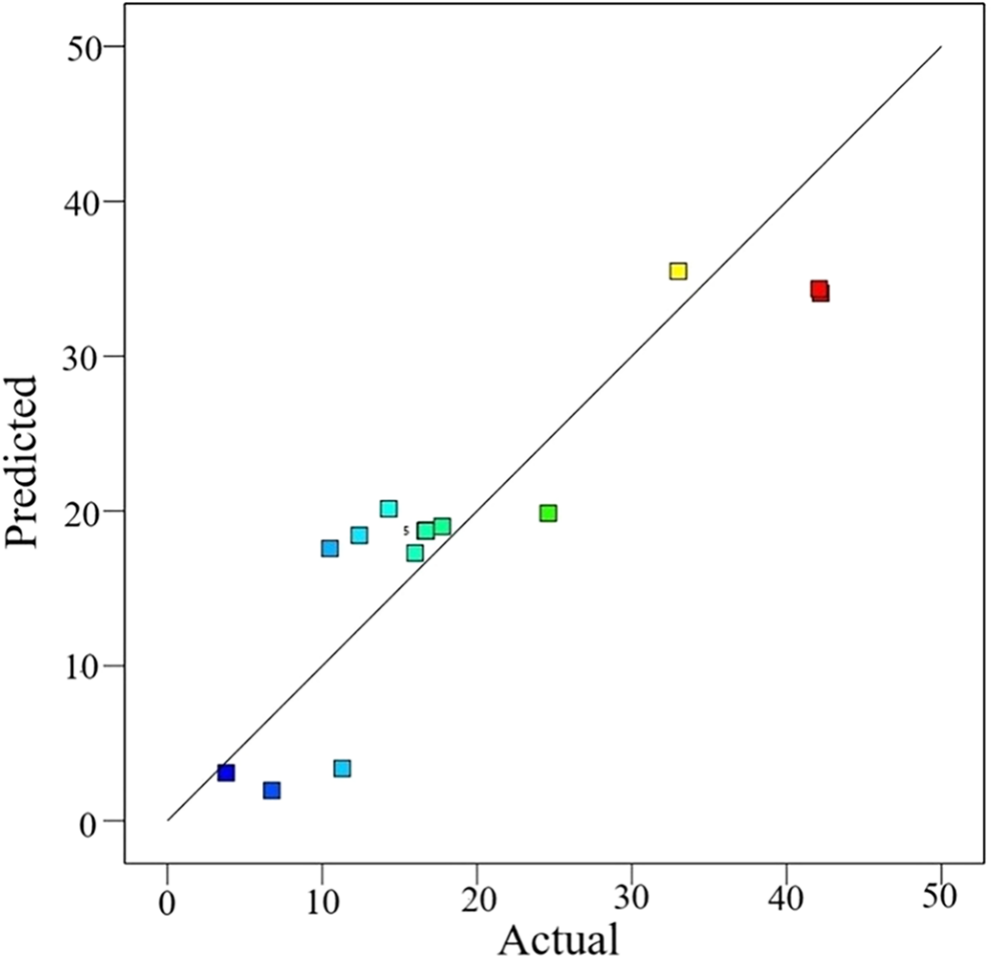
Predicted and actual removal rate values.

The perturbation plot presented in Figure 13 shows a comparison of three different parameters (adsorbent amount, dye concentration, and adsorption time). While the removal is positively proportional to the amount of adsorbent and time, it is negatively proportional to the dye concentration. The linear effect of the variables on the removal is more clearly observed in the perturbation plot.

**Figure 13:**
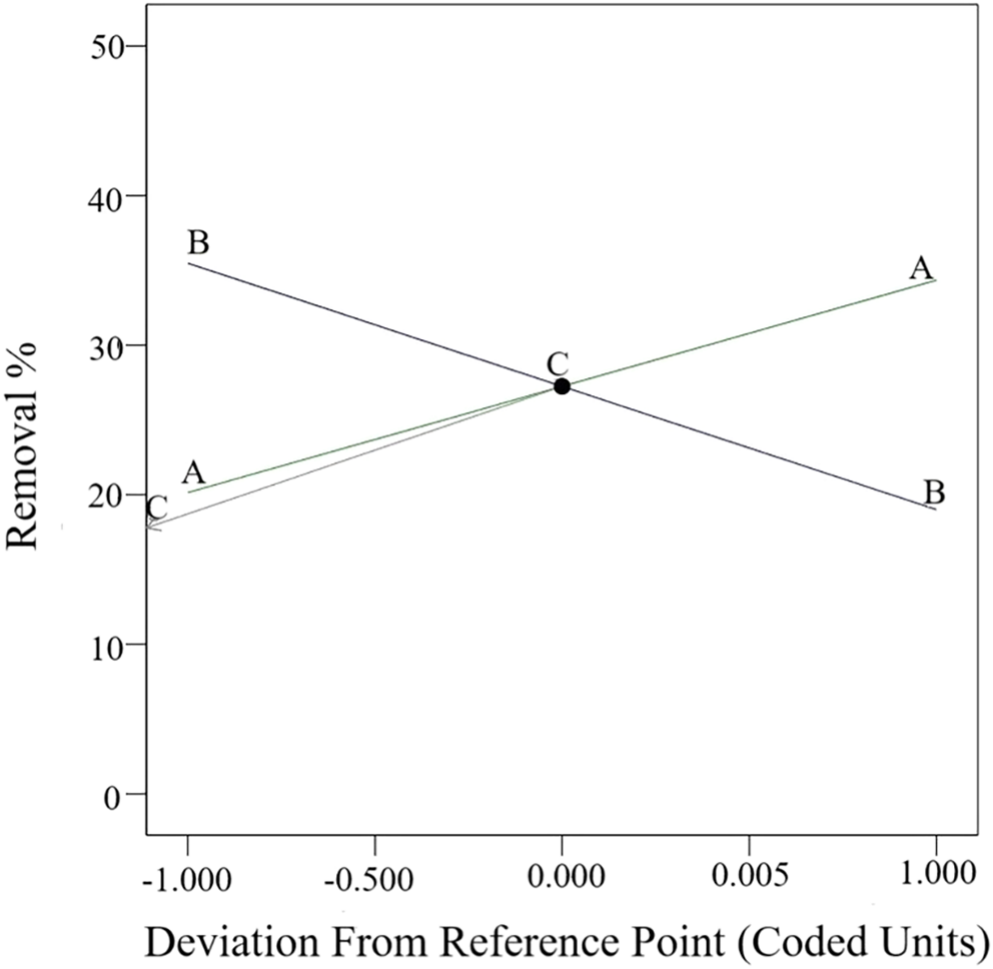
Perturbation graph.

In the 3D surface graph illustrated in Figure 14, when the adsorbent amount was raised from 30 to 50 mg, a sharp increase in the removal was seen. Conversely, when the dye concentration was raised from 10 to 20 mg/L, a dramatic decrease in the removal rate was observed. These changes are more clearly shown in different colors.

**Figure 14:**
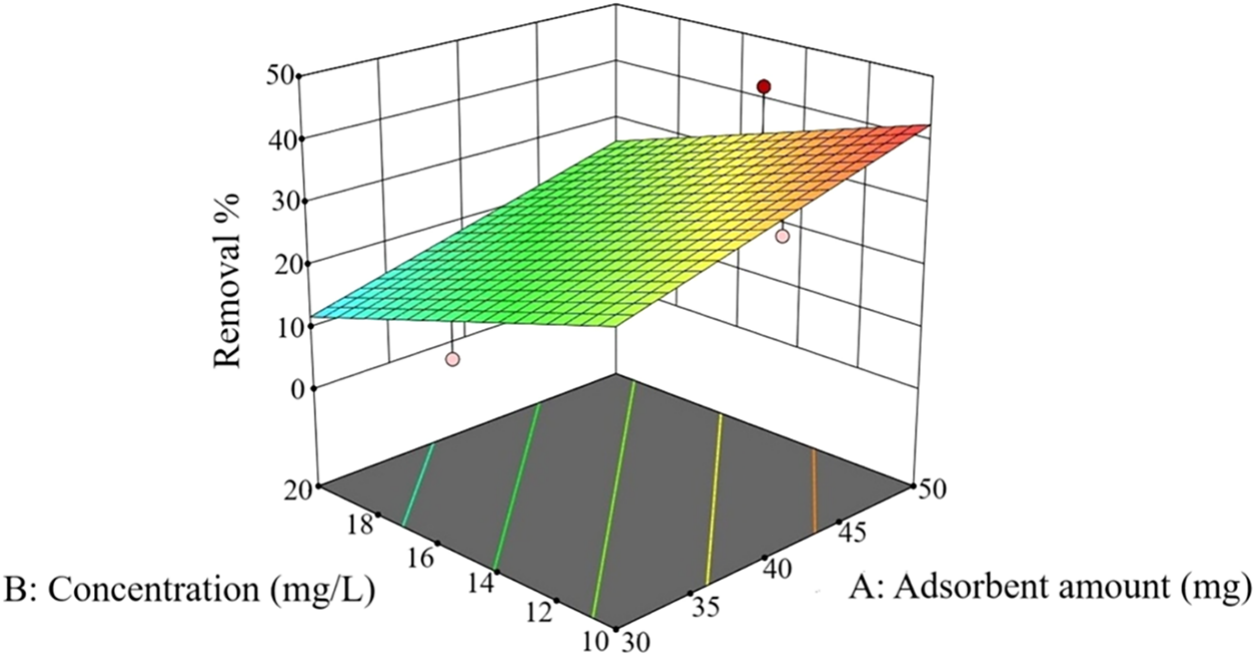
3D surface graph.

In Figure 15, the 2D contour graph drawn based on the values obtained from the experimental conditions suggested by the response surface method is shown for A, 10 mg; B, 10 mg/L; and C, 180 min. As the amount of adsorbent increases, the green color on the graph changes to warmer tones; thus, the amount of adsorbent is a key variable for the experiment. Under these conditions, desirability is 1.

**Figure 15:**
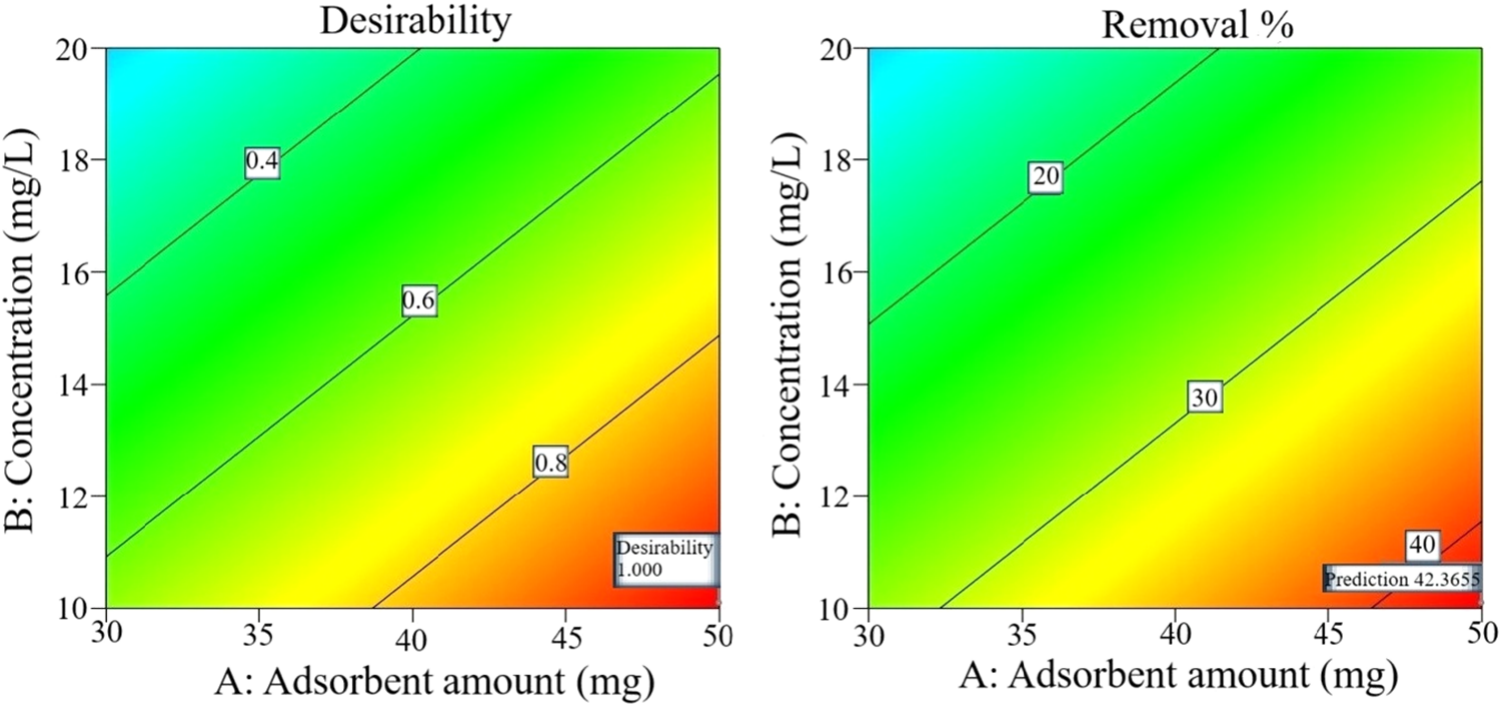
2D contour graph.

### Comparison with Literature

A comparison of malachite green adsorption by the adsorbent studied with those of other adsorbents in the literature is given in Table 10.

**Table 10:** Comparison of Adsorbent Developed with Adsorbents in the Literature

nanoadsorbent	dye concentration, adsorbent dosage, pH, temperature, time	removal %	reference
magnetic laccase nanoflowers encapsulated Fe_3_O_4_	10 mg/L	99	^56^
	–		
	pH: 4.5		
	–		
	15 min		
magnetic Fe_3_O_4_ functionalized epoxy-triazinetrione nanoadsorbent	–	57	^57^
	15 mg		
	pH: 10		
	40 °C		
	20 min		
reduced graphene oxide	60 mg/L	96.32	^58^
	40 mg		
	pH: 8		
	30 °C		
	20 min		
zein biopolymer and graphene oxide nanofibrous composite	100 mg/L		^59^
	8 mg		
	pH: 6		
	25 °C		
	–		
cellulose nanofiber and silver nanoparticles composite	100 mg/L	78	^60^
	10 mg		
	pH: 8		
	30 °C		
	10 min		
functionalized nanoporous silica	10 mg/L		^61^
	20 mg		
	pH: 6.5		
	–		
	60 min		
carboxymethyl cellulose-grafted poly(acrylamide) and montmorillonite nanocomposite hydrogel	10 mg/L		^62^
	–		
	pH: 7		
	30 °C		
	240 min		
polypyrrole–iron oxide–seaweed nanocomposite	150 mg/L	99.79	^63^
	50 mg		
	pH: 7		
	–		
	40 min		
bioglass and biopolymer alginate nanocomposite	10 mg/L	47.5	this study
	50 mg		
	pH: 6.46		
	25 °C		
	180 min		
	shaking speed: 300 rpm		
bioglass and biopolymer alginate nanocomposite	10 mg/L	90	this study
	50 mg		
	pH: 6.46		
	25 °C		
	180 min		
	shaking speed: 600 rpm		

## Conclusions

In this study, a new biobased adsorbent was developed to remove the toxic dye and disinfectant, malachite green, from water. Malachite green adsorption from synthetic wastewater was performed with the adsorbents. The effects of adsorbent amount, dye concentration in aqueous solution, and time on the adsorption process using bioadsorbent formed from bioglass-added alginate were investigated. As a result of statistical modeling performed with Box–Behnken in the response surface method, it was observed that the most effective variable in the adsorption process was time. Adsorption efficiency changed negatively with the dye concentration and positively with the adsorbent amount. Isotherm studies indicated that adsorption equilibrium data were compatible with the Langmuir isotherm data. Kinetic model studies demonstrated that the relationship between adsorption rate and time could be explained by the Pseudo-first-order kinetic model. According to thermodynamic studies, it was reported that adsorption decreased with temperature. The dye with a positive charge was adsorbed by the adsorbent with negative groups. The developed bionanocomposite adsorbent shows promise for dye removal from water.
